# New Insights into the Pathogenesis of Alcohol-Induced ER Stress and Liver Diseases

**DOI:** 10.1155/2014/513787

**Published:** 2014-04-29

**Authors:** Cheng Ji

**Affiliations:** USC Research Center for Liver Disease, Department of Medicine, Keck School of Medicine of USC, University of Southern California, 2011 Zonal Avenue, HMR-101, Los Angeles, CA 90089, USA

## Abstract

Alcohol-induced liver disease increasingly contributes to human mortality worldwide. Alcohol-induced endoplasmic reticulum (ER) stress and disruption of cellular protein homeostasis have recently been established as a significant mechanism contributing to liver diseases. The alcohol-induced ER stress occurs not only in cultured hepatocytes but also * in vivo*  in the livers of several species including mouse, rat, minipigs, zebrafish, and humans. Identified causes for the ER stress include acetaldehyde, oxidative stress, impaired one carbon metabolism, toxic lipid species, insulin resistance, disrupted calcium homeostasis, and aberrant epigenetic modifications. Importance of each of the causes in alcohol-induced liver injury depends on doses, duration and patterns of alcohol exposure, genetic disposition, environmental factors, cross-talks with other pathogenic pathways, and stages of liver disease. The ER stress may occur more or less all the time during alcohol consumption, which interferes with hepatic protein homeostasis, proliferation, and cell cycle progression promoting development of advanced liver diseases. Emerging evidence indicates that long-term alcohol consumption and ER stress may directly be involved in hepatocellular carcinogenesis (HCC). Dissecting ER stress signaling pathways leading to tumorigenesis will uncover potential therapeutic targets for intervention and treatment of human alcoholics with liver cancer.

## 1. Introduction

The endoplasmic reticulum (ER) is an essential organelle of eukaryotic cells functioning in secretory protein synthesis and processing, lipid synthesis, calcium storage/release, and detoxification of drugs. The ER ensures correct protein folding and maturation. Unfolded proteins are retained in the ER and targeted for retrotranslocation to the cytoplasm for rapid degradation. Under normal physiological conditions, there is a balance between the unfolded proteins and the ER folding machinery. Disruption of the balance results in accumulation of unfolded proteins, a condition termed ER stress [1–5]. The ER stress triggers the unfolded protein response (UPR), which attenuates protein translation, increases protein folding capacity, and promotes degradation of unfolded proteins, thus restoring ER homeostasis. However, prolonged UPR leads to an attempt to delete the cell causing injuries. Molecular chaperones such as the glucose-regulated protein 78 (GRP78/BiP) interact with three ER membrane resident stress sensors: inositol-requiring enzyme-1 (IRE1*α*), transcription factor-6 (ATF6), and PKR-like eukaryotic initiation factor 2*α* kinase (PERK), and play a vital role in maintaining the protein homeostasis inside the ER [1–5]. Many human diseases such as metabolic syndrome, neurodegenerative diseases, alcohol-induced organ disorders, and inflammatory diseases involve ER stress and impaired UPR signaling [1–7]. Increasing evidence supports ER stress as a key mechanism in alcohol-induced liver disease (ALD), a disease that affects over 140 million people worldwide. Potential molecular mechanisms underlying alcohol-induced ER stress in major organs including liver, brain, pancreas, lung, and heart have been discussed previously [8–10]. In this review, I will focus on updates and new insights into the pathogenesis of alcohol-induced ER stress and discuss an emerging role of alcohol-induced ER stress in liver tumorigenesis and hepatocellular carcinogenesis.

## 2. Multiple Mechanisms for Alcohol-Induced Hepatic ER Stress

**Figure 1:**
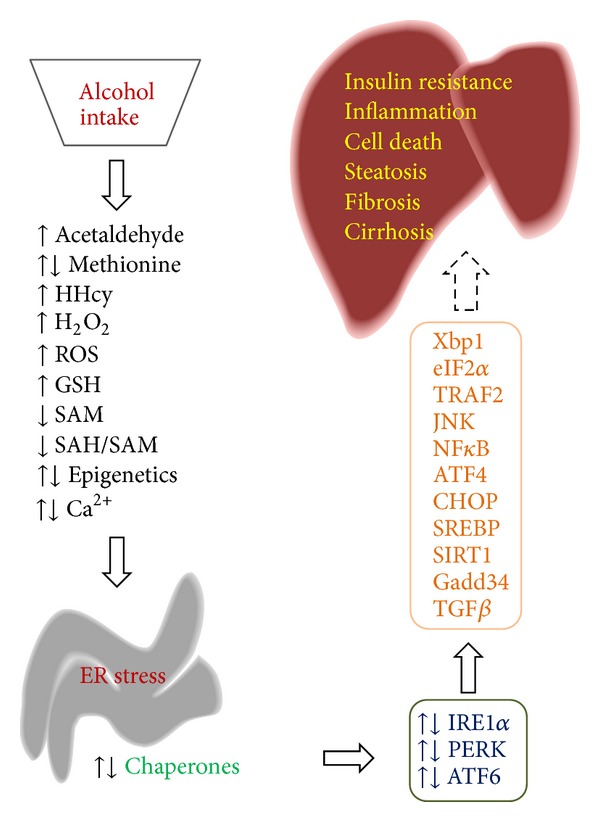
Identified molecular mechanisms for alcohol-induced endoplasmic reticulum stress and hepatic injuries. See the context for details.

Alcohol is mainly metabolized in the liver and liver cells are rich in ER, which assume synthesis of a large amount of secretory and membrane proteins. The UPR plays a pivotal role in maintaining ER homeostasis in the liver under both physiological and pathological conditions [4, 5, 9]. In the early 80s, stress-induced ER damages in the liver were observed in ultrastructural, morphological, and histological studies [43, 44]. However, little was known then about occurrence and mechanisms of alcohol-induced ER stress. A role of ER in alcohol metabolism began to be recognized as NADH from the hepatic alcohol oxidation by alcohol dehydrogenase (ADH) was also found to support microsomal alcohol oxidations [43–46]. The inducible microsomal ethanol oxidizing system (MEOS) is associated with ER proliferation and concomitant induction of cytochrome P4502E1 (CYP2E1) in rats and in humans [45, 46]. Free radical release, as a consequence of CYP2E1 activities in the ER and subsequent oxidative stress, and lipid peroxidation generally contribute to ALD. However, alcohol-induced ER stress response (AERR) that involves the UPR was not recognized until recently. Molecular evidence for an impaired UPR was first found in the mice with chronic intragastric alcohol infusion (CIAI) (Figure 1; Table 1) [11]. Alterations of some ER stress markers: GRP78, GRP94, CHOP (C/EBP homologous protein), and BAD (the Bcl-2-associated death promoter), in DNA microarrays were associated with severe steatosis, scattered apoptosis, and necroinflammation. SREBP-1c (sterol regulatory element-binding protein-1c) was found to be a strong candidate linking ER stress to alcoholic fatty liver, because SREBP-1c knockout mice were protected against triglyceride accumulation [12]. CHOP was found to be a key factor in AERR-caused cell death, as knocking out CHOP resulted in minimal alcohol-induced apoptosis in the liver [13].

Upstream of ER stress, altered methionine metabolism, and elevated homocysteine were initially proposed to be responsible for AERR because alcohol-induced hyperhomocysteinemia (HHcy) is often seen in rodents and humans [47–50] and homocysteine is known to modify proteins biochemically [8, 9, 11]. A few lines of molecular evidence support the methionine/homocysteine mechanism. First, betaine is a methyl donor for remethylation of homocysteine to methionine catalyzed partially by betaine-homocysteine methyltransferase (BHMT). Simultaneous betaine feeding or transgenic expression of BHMT in CIAI mice decreased the elevated homocysteine and abrogated AERR in parallel with decreased ALT and amelioration of ALD [11, 14, 15]. Second, an intragastric infusion with both high fat and alcohol induced moderate obesity and much severe ALD [16], which resulted from synergistic effects of an accentuated ER stress by the alcohol-induced HHcy in combination with mitochondrial stress, nitrosative stress, and adiponectin resistance. Third, rats with CIAI do not have a significant HHcy [17]. Consequently, the rat animals have a minimal ER stress response and are more resistant to ALD, which correlates with a significant induction of BHMT. Fourth, in a study with 14 inbred mouse strains with CIAI, profound differences in ALD were observed among the strains in spite of consistently high levels of urine alcohol [18]. ER stress related genes were induced only in strains with the most liver injury, which were closely associated with expression patterns of methionine metabolism-related genes and plasma homocysteine levels. Thus, abnormal protein modifications by excessive homocysteine as a result of aberrant one-carbon metabolism [18] and methionine deficiency [4] are likely responsible for the alcoholic ER stress and UPR in CIAI mice that lack a sufficient upregulation of BHMT.

However, other causes for the alcoholic ER stress are present in the CIAI model. For instance, in a study with CIAI rats to examine effects of selective inhibition of CYP2E1 on the development of alcoholic fatty liver [19], liver triglycerides were lower. ER stress indicated by the ER stress marker TRB3 (a mammalian homolog of* Drosophila* tribbles functions as a negative modulator of protein kinase B) was increased after ethanol and was further increased upon inhibition of CYP2E1 or overall ethanol metabolism. This suggests a contributing role of alcohol metabolites, for example, acetaldehyde, or oxidants to the alcoholic ER stress response. In another study with cystathionine *β* synthase (CBS) heterozygous mice treated with CIAI [20], steatohepatitis was accompanied with upregulations of hepatic ER stress components including GRP78, ATF4 (activating transcription factor 4), CHOP, and SREBP-1c and negatively correlated with S-adenosylmethionine (SAM) to S-adenosylhomocysteine (SAH) ratio. AERR was associated with a decrease in levels of suppressor chromatin marker trimethylated histone H3 lysine-9 (3meH3K9) in the promoter regions of the ER stress markers. Similarly, epigenetic mechanism for AERR might also occur in human alcoholics, as DNA hypermethylation of the promoter of HERP (homocysteine-induced ER protein) gene downregulates its mRNA expression in patients with alcohol dependence [51].

## 3. Diverse Models and Species with Alcohol-Induced ER Stress

AERR occurs not only in the aforementioned CIAI models but also in other chronic or acute models/systems (Table 1), which have been providing additional insights into AERR and ALD. In micropigs fed alcohol orally [21], liver steatosis and apoptosis were shown to be accompanied by increased mRNA levels of CYP2E1 and selective ER stress markers. Folate deficiency appeared to be responsible for the ER stress and injury. In mice, however, oral alcohol feeding* ad libitum* does not usually result in HHcy as remarkable as seen in the CIAI mice. Correspondingly, the degree of AERR and subsequent liver injury may depend on additional genetic and/or dietary factors. For instance, in the mice with liver specific deletion of GRP78/BiP [22], a robust ER stress response was observed at moderate oral alcohol doses (e.g., 4 g/kg), which was accompanied by much aggravated hepatosteatosis and hepatic fibrosis. Thus, compared to the homocysteine-ER stress mechanism, the liver BiP deletion represents a genetic predisposition that unmasks a distinct mechanism by which alcohol induces ER stress, one that is largely obscured by compensatory changes in normal animals or presumably in the majority of human population who have low-to-moderate drinking [8, 22]. The effect of genetic predisposition on AERR and hepatic injury is also observed in a recent study using mice with low alcohol-induced plasma homocysteine and deficient in the acid sphingomyelinase (ASMase) [23]. Strong AERR and enhanced susceptibility to lipopolysaccharides (LPS) or concanavalin-A were present in ASMase ^−^/^−^ mice fed alcohol orally, indicative of a mitochondrial effect on AERR. In addition, in iron overloaded mice deficient in the hemochromatosis gene (Hfe^−^/^−^), cofeeding* ad libitum* with alcohol and a high-fat diet (HFD) led to profound steatohepatitis and fibrosis [24, 25]. XBP1 splicing, activation of IRE-1*α* and PERK, and increased CHOP protein expression were associated with impaired autophagy response, suggesting that preconditioning with iron overloading may modulate AERR and promote liver injury through interacting with other adaptive or compensatory mechanisms.

Alternatively, the contributing role of ER stress to ALD in oral feeding models could be secondary. This is indicated by a time-course study with a mouse model of early-stage ALD [26]. Mice with oral alcohol feeding exhibited significant hepatic steatosis and elevated plasma ALT values. At 1 to 2 weeks after alcohol feeding, oxidative stress indicated by 4-hydroxynonenal- (4-HNE-) modified proteins was increased, whereas hepatic glutathione (GSH) levels were significantly decreased as a consequence of decreased CBS activity, increased GSH utilization, and increased protein glutathionylation. Except for 4-HNE adduction to the ER disulfide isomerase (PDI), significant upregulations of other ER markers and SREBP pathways were not detected* in vivo* during the same early period of alcohol feeding [26, 27]. Although the actual blood alcohol levels were not measured in this study, which might not reach a critical point and vary widely among individual mice at a liberal access to alcohol, the results may suggest a secondary role of AERR in this early ALD model. Thus, interplay or cross-talk between AERR and other stresses might be critical in ALD. This notion is supported by a few most recent reports, which appears more evident in cell or animal models with acute alcohol exposure. First, cell death is not readily observed in acute ethanol intoxication. However, in a perfused rat liver system, downregulation of GRP78 and activation of c-Jun N-terminal kinase (JNK) and protein kinase B (PKB/Akt) were enhanced by a cotreatment of acute ethanol with a classical inhibitor of ADH, and an antioxidant addition reduced the activation of JNK and cell death [28]. High concentrations of the pharmacological ER stress-inducing agents such as tunicamycin or brefeldin A activate JNK and inhibit mitochondrial respiration and cell death in hepatocytes [52]. Mitochondrial respiration has been shown to play an adaptive role in ALD [53]. Thus, ethanol metabolites and/or impaired mitochondrial functions may complicate AERR. Second, the mice with liver specific GRP78 deletion are sensitized to a variety of acute hepatic disorders by alcohol, a high-fat diet, anti-HIV drugs, or toxins [22]. HIV protease inhibitors inhibit the ER Ca^2+^ATPase (SERCA) and modulate calcium homeostasis in mice and primary human hepatocytes, which aggravates AERR and ALD [29]. Alcohol-induced LPS impairs UPR promoting rat liver cirrhosis [30]. Third, the interferon regulatory factor 3 (IRF3) is activated early by ER stress in mice fed alcohol either orally or intragastrically, which involves an ER adaptor, the stimulator of interferon genes (STING) [31]. Independent of inflammatory cytokines and Type-I interferons (IFNs), IRF3 exerts its pathogenic role in ALD through causing apoptosis of hepatocytes, which strongly suggests that AERR pathways and the LPS-TLR4 (toll-like receptor 4) pathways [54] are parallel or equally important in initiating ALD.

In addition to rodents, AERR has also been found in other species including human alcoholics (Table 1). Zebrafish larvae represent an alternative vertebrate model for studying AERR and ALD because their liver possesses the pathways to metabolize alcohol that can be simply added to the water, that is, acute alcohol [32]. AERR is present in alcohol-treated zebrafish, which may also interact with other pathological factors. Upon alcohol challenge, zebrafish larvae developed signs of acute ALD, including hepatomegaly and steatosis. Further, the ER stress response appeared much robust in zebrafish deficient in the CDP-diacylglycerol-inositol 3-phosphatidyltransferase (CDIPT) that primarily locates on the cytosolic aspect of the ER [33]. Thus, integrity of the ER or alcohol metabolism might be necessary for AERR [34]. In supporting this, in the species* Caenorhabditis elegans* without a liver for alcohol digestion/metabolism, little AERR has been detected [35]. The most important and clinically relevant studies regarding AERR are from human cells and patients. AERR is reported in human monocyte-derived dendritic cells (MDDC) [36], HepG2 cells expressing human CYP2E1 [37], and primary human hepatocytes [29]. Oxidative stress resulted from the function of CYP2E1 and/or interactions with other drugs contributing to AERR in the human cells. However, cultured human cell models may not reflect the complexity of the response* in vivo*. For instance, it was reported that, upon alcohol exposure, VL-17A cells metabolized alcohol which caused ER fragmentation inside the cells, but little activation of UPR target genes was detected [38]. Nevertheless, striking upregulation of multiple ER stress signaling molecules was detected in human patients with ALD (Table 1) [39–42], which is correlated with deregulated lipid metabolism, ceramide accumulation, and impaired insulin signaling, indicating that AERR is an integrated part of pathogenesis of ALD in human alcoholics.

## 4. Emerging Role of AERR in Liver Tumorigenesis and HCC

It has been well accepted that the UPR is a double-edged response because both adaptive survival and eliminative apoptosis can be induced by UPR components [1–6]. It is beneficial or prosurvival if it happens transiently or lasts for a short period of time, whereas it is detrimental or deadly if it is prolonged. Recent studies indicate that the UPR is associated with solid tumor development in many types of tissues or organs including the liver [55, 56]. Since the microenvironments of solid tumors are generally hypoxic, acidic, and nutrient deficient [57, 58], which individually or collectively favor activation of ER stress response, it is conceivable that the UPR is persistently present during tumorigenesis. The question is how the cancer cells evade cell death from the prolonged UPR. Emerging evidence suggests that cancerous cells could modify and perturb the ER stress-associated cell death signaling, which permits survival and growth. For instance, the master regulator UPR, GRP78, plays a dual role in tumor cells [22, 59]. It controls early tumor development through suppressive mechanisms such as the induction of cell cycle arrest or tumor dormancy upon PERK activation [60]. On the other hand, at more advanced stages of tumor progression, during which cells are exposed to more severe stressors, GRP78 suppresses caspase 7 activation and interacts with ER stress-induced protein chaperones such as clusterin to promote cell survival and further tumor development [61]. The PERK-eIF2*α*-ATF4 pathway is often activated by the hypoxic condition in solid tumors [62–64], which activates angiogenic genes, vascular endothelial growth factor (VEGF), type 1 collagen inducible protein, and autophagosome components such as LC3, ensuring cell survival over hypoxia-induced ER stress [65–67]. Prolonged expression and activation of ATF6 increase the Rheb-mTOR signaling pathway and also enhance tumor cell survival [68]. In addition, the IRE1*α*-Xbp1 pathway interacts with antiapoptotic Bcl-2 family members and the sigma-1 receptor, which is often increased in many human cell lines [69–72]. Therefore, impaired and/or prolonged UPR has a high potential to modulate cell fates and differentiations towards tumorigenesis.

Alcohol intake increasingly contributes to mortality from liver cancer in humans [73–76]. However, the mechanisms by which alcohol exerts its carcinogenic effect are largely unknown and currently there is no effective treatment. Considering that several lines of evidence indicate that polymorphic responses of major ER chaperones to alcohol and other stressors are associated with hepatocellular carcinogenesis in human populations [77–82], it is not unusual to find a role of AERR in HCC. In fact, we recently found spontaneous hepatocellular adenomas- (HCA-) like tumors in aged female mice with a liver specific BiP deletion and under constitutive ER stress [22, 59, 83]. Active ATF6, CHOP, GSK3*β*, and Creld2 (cysteine rich with EGF-like domains 2) were increased in the knockout, indicative of continuous ER stress response. None of p53, HNF1*α*, or GP130 was significantly changed compared between wild type and knockout. *β*-Catenin was slightly decreased. Interestingly, cyclin D was specifically reduced in the tumor portion of the knockout mice. Since most liver tumors were found in female knockouts, expression of receptors for sex hormones such as estrogen receptors, ER*α* and *β*, and androgen receptor, AR*α*, was examined. Three variants of ER*α* were detected in the liver, and their molecular sizes are 66 kD, 46 kD, and 36 kD, respectively [83]. The ER*α* variant 36 kD was remarkably increased in the tumor portion of the knockout liver. In contrast, there were no significant changes in the expression of ER*β*, AR*α*, cyclin E, or cyclin G. These findings revealed thatinhibition of cyclin D and overexpression of ER variant 36 kD are associated with the tumor development in the female knockouts under constitutive ER stress [83]. Furthermore, the tumors are highly malignant in mice with additional stresses such as high-fat diet or alcohol intake [83, 84]. The pathways of ERK1/2, Stat3, and p38 were activated, which are known to promote HCC progression [85, 86].

**Figure 2:**
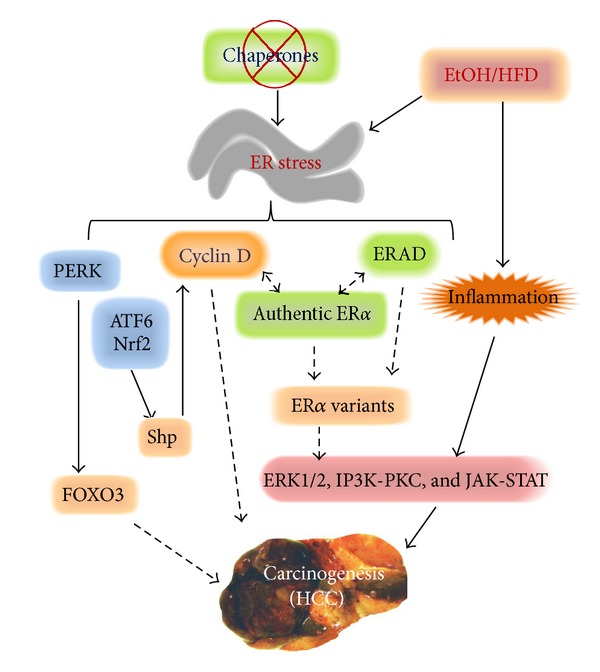
Proposed model depicts novel endoplasmic reticulum (ER) stress mechanisms linking alcohol (EtOH) and/or high-fat diet (HFD), cyclin D, ERAD, estrogen receptor *α* (ER*α*) variants, FOXO3, and Shp with hepatocellular carcinogenesis (HCC). Solid lines represent established pathways based on the literature; dashed lines represent emerging mechanisms under investigations. See the context for details.

The constitutive ER stress-induced spontaneous liver tumors that are dominant in female animals are similar to human HCA [87–90], which are of clinical relevance since about 80% of HCA cases are from women taking oral contraceptives for years [90, 91]. The pathogenesis of HCA is not completely understood due to its heterogeneity. Known potential causesfor human HCA are mutations in HNF1*α*, *β*-catenin, GP130, or chronic inflammation [87–93]. Hepatocellular protein homeostasis has rarely been noticed to be a potential mechanism for HCA development. Thus, the above findings on cyclin D and ER*α* variants may reveal a novel ER stress mechanism for HCA for several reasons (Figure 2). First, the* in vivo* inhibition of cyclin D expression upon ER stress in knockouts is consistent with an earlier study, which demonstrated that activation of the UPR in mouse NIH 3T3 fibroblasts with tunicamycin led to a decline in cyclin D and subsequent G(1) phase arrest [94–96]. Second, increased expression of cyclin D is usually associated with proliferation in other systems [97]. However, a number of studies have shown many new roles of cyclin D [98] and a surprising lack of the correlation of increased cyclin D with proliferation in tumors [99, 100]. For instance, in one subtype of human breast carcinoma, cyclin D1 protein expression was absent in the noninvasive cells [101, 102]. Similarly, a loss of cyclin D did not inhibit the proliferative response of mouse liver to mitogenic stimuli [103] and mRNA levels of cyclin D1 were downregulated in patients with HCC [104]. Most recent molecular evidence further supports this ER stress-cyclin D-tumorigenesis mechanism. Nrf2 (the nuclear factor erythroid 2-related factor 2) activities are associated with aging [105]. ER stress activates Nrf2 and ATF6, both of which regulate the orphan nuclear receptor, Shp (small heterodimer partner) which acts as a transcriptional corepressor modulating cyclin D1 and subsequent hepatic tumorigenesis [106, 107]. The ER stress sensor PERK has been shown to phosphorylate the Forkhead transcription factor 3 (FOXO3) [108] and suppressed FOXO3 exacerbates alcoholic hepatitis and insulin resistance impairing cyclin D function promoting HCC [109–111]. Thus, abnormal functions of cyclin D under ER stress conditions most likely disturb liver cell proliferation (Figure 2). Third, since the authentic ER*α*66 interacts with cyclin D physically [100, 101], the hepatic ER*α* variants may result from an unbalanced long-term molecular interaction between ER*α* and the suppressed cyclin D under ER stress (Figure 2). Alternatively, the ER*α* variants may be produced from an incomplete protein processing/maturation of ER*α* by an impaired ER-associated degradation (ERAD). Components of ERAD are indeed altered in the BiP knockout mouse models under constitutive UPR [22, 59, 83, 84], and there is a report indicating that an activation of the Xbp1-Hrd1 (an E3 ubiquitin ligase also called synoviolin) branch by the UPR facilitates Nrf2 ubiquitylation and degradation during liver cirrhosis [112]. Similarly, ER*α* could also be a target of the impaired ERAD. Fourth, considering that ER*α* gene polymorphism is associated with risk of HBV-related acute liver failure [113] and a switch from the authentic ER*α* to a predominant expression of ER*α*36 is associated with development and progression of HCC [114–116], the hepatic ER*α* variants could also play an important role in alcohol and ER stress-associated HCC. The tumorigenic signaling downstream of cyclin D and ER*α* variants can be activations of the ERK1/2, IP3K-PKC, or JAK-STAT pathways [117]. Overexpressed ER*α*36 has been associated with activation of these pathways and carcinogenesis in other systems such as breast cancer and gastric cancer [118–122]. In the liver, activations of ERK1/2 and JAK-STAT pathways were observed in the BiP knockout mice fed alcohol and high-fat diet [83, 84]. Finally, studies on hepatoma cell lines, HCC tissues, and animal models of HCC suggest a possible role of sex hormones and their receptors in HCC pathogenesis [123]. A male prevalence of HCC is often observed in young and middle aged patient populations in certain regions exposed to additional HCC risk factors [124]. However, the male prevalence of HCC tends to diminish in aged human populations [125] as well as in aged animals fed alcohol [83, 84]. Therefore, alcohol-induced ER stress and cell cycle impairment may exert specific effects on aging, hepatic expression of estrogen receptors, and subsequent tumorigenesis in females.

## 5. Conclusive Remarks

Alcohol-induced hepatic ER stress occurs in the liver of many species including human alcoholics, which has recently been established as an important mechanism for both acute and chronic alcohol-induced liver pathogenesis and disease development. Multiple factors commonly associated with alcohol consumption such as acetaldehyde, oxidative stress, excessive homocysteine, toxic lipid species, increased SAH, aberrant epigenetic modifications, disruption of calcium homeostasis, and insulin resistance induce ER stress response individually or collectively. However, the precise contribution of each of the factors to the ER stress induction is not clear and their importance to ALD may depend on doses, duration and patterns of alcohol exposure, presence or absence of genetic and environmental factors, cross-talks with other pathogenic pathways, and liver disease stages. The UPR, as an integrated part of liver physiology and pathology like the immune response, may occur more or less all the time during alcohol consumption, which attempts to restore ER homeostasis and protect against ALD. However, this adaptive protection is not without detrimental consequences. Prolonged UPR leads to excessive deletion of the damaged hepatocytes or cell cycle arrest, which triggers inflammatory response or interrupts normal cellular processes causing profound injuries. Emerging evidence by us and others indicates a direct involvement of long-term alcohol and constitutive ER stress in liver tumorigenesis and hepatocellular carcinogenesis. The ER stress and malfunctioning of cyclin D-caused cell cycle arrest are a well-established molecular mechanism, and the surprise overexpression of estrogen receptor *α* variants under constitutive UPR may result from a mal-targeting of protein processing and turnover by aberrant ERAD, which reflect complexity and depth of prolonged UPR-mediated pathogenesis. Thus, liver tumorigenesis by alcohol and ER stress may involve not only cyclin D, ER*α* variants, ERK1/2, PKC, and STAT pathways, but also other cell cycle targets such as IL-6, p21, p27, and CDK, other pathways such as Src/EGFR, PTEN-TGF*β*, and insulin/IGF, and epigenetic regulations such as miRNAs targeting the UPR components. In addition, liver progenitor cell activation by alcohol may contribute to the malignant transformation of nonmalignant tumors developed under long-term ER stress [22, 59]. Future work should be directed to define the ER stress mechanisms leading to HCC and to develop multiple therapeutic approaches to target ER stress in human alcoholics with HCC.

## Figures and Tables

**Table 1:** Alcohol-induced endoplasmic reticulum stress (AERR) and injuries occur in many species.

Experimental system	Cause	Injury	Remark	Reference
Chronic intragastric infusion				
Mouse	Hyperhomocysteinemia	Necroinflammation	Mouse strain difference	[11–13]
	Methionine deficiency	Apoptosis	Rat and mouse difference	[14–18]
	Acetaldehyde adducts	Fatty liver	Synergy with obesity	[19]
Rat	High SAH	Fibrosis		[20]
	Low SAM/SAH			
	Epigenetic alterations			
Chronic oral feeding				
Micropig	Folate deficiency	Steatosis		[21]
Mouse	Chaperone deficiency	Apoptosis	Interaction of alcohol with	[22]
	Synergy with HFD/drugs	Fibrosis	anti-HIV/HCV drugs	[23]
	Excess iron	Cirrhosis	Involvement of autophagy	[24, 25]
	Oxidative stress		Oxidative stress precedes AERR	[26, 27]
Acute alcohol exposure				
Liver perfusion	Acetaldehyde, ROS	Fat accumulation	Role of alcohol metabolites in AERR	[28]
Mouse gavage	Synergy with drugs	Apoptosis		[22]
	Ca^2+^ homeostasis	Fibrosis	AERR parallels LPS-TLR4	[29, 30]
	Inflammation		Suppressed UPR	[31]
Zebrafish	CDIPT deficiency	Hepatomegaly		[32–34]
Nematode	Not known	Not characterized	No AERR without the liver	[35]
Alcohol treated cells				
Human cells	ROS	Apoptosis	Basal ER stress in HepG2	[36–38]
	Excessive homocysteine	Steatosis		
Patient liver biopsies				
Human alcoholics	Toxic lipid species	Apoptosis	Clinical relevance	[39–42]
	Oxidative stress	Steatohepatitis	Role of mitochondrial	
	Insulin resistance	Fibrosis/cirrhosis	Dysfunctions in AERR	

## Abbreviations

ADH: Alcohol dehydrogenase
AERR: Alcohol-induced ER stress response
ALD: Alcohol-induced liver disease
AR*α*: Androgen receptor *α*
ASMase: The acid sphingomyelinase
ATF 4 or 6: Activating transcription factor 4 or 6
BAD: The Bcl-2-associated death promoter
BiP: Binding immunoglobulin protein also known as 78 kDa glucose-regulated protein (GRP78)
BHMT: Betaine-homocysteine methyltransferase
CBS: Cystathionine *β* synthase
CDIPT: CDP-diacylglycerol-inositol 3-phosphatidyltransferase
CDK: Cyclin-dependent kinase
CHOP: C/EBP homology protein 10
CIAI: Chronic intragastric alcohol infusion
Creld2: Cysteine rich with EGF-like domains 2
EGFR: Activation of the epidermal growth factor receptor
ER: Endoplasmic reticulum
ER*α*: Estrogen receptors *α*
ERAD: ER-associated degradation
ERK1/2: Extracellular signal-regulated protein kinases 1 and 2
FOXO3: The forkhead transcription factor
HCA: Hepatocellular adenomas
HCC: Hepatocellular carcinogenesis
HERP: Homocysteine-induced ER protein
HFD: High-fat diet
HHcy: Hyperhomocysteinemia
4-HNE: 4-Hydroxynonenal
HNF1*α*: Hepatocyte nuclear factor 1*α*
Hrd1: An E3 ubiquitin ligase also called synoviolin
GADD34: Growth arrest and DNA damage-inducible protein 34
GSH: Glutathione
GP130: Glycoprotein 130
GSK3*β*: Glycogen synthase kinase 3*β*
IGF: Insulin-like growth factor
IRE1*α*: Inositol-requiring enzyme-1*α*
IRF3: The interferon regulatory factor 3
JAK: Janus kinase
JNK: c-Jun N-terminal kinases
LC3: Microtubule-associated protein 1A/1B-light chain 3
LPS: Lipopolysaccharides
Mito: Mitochondria
MDDC: Human monocyte-derived dendritic cells
MEOS: The inducible microsomal ethanol oxidizing system
mTOR: Mammalian target of rapamycin
NF*κ*B: Nuclear factor *κ*B
PDI: The ER disulfide isomerase
Nrf2: The nuclear factor erythroid 2-related factor 2
PGC1: PPAR*γ* coactivator 1
PERK: PKR-like ER-localized eIF2*α* kinase
PKC: Protein kinase C
PTEN: Phosphatase and tensin homolog
ROS: Reactive oxygen species
SAH: S-Adenosylhomocysteine
SAM: S-Adenosylmethionine
SERCA: ER calcium transport ATPase
Shp: Small heterodimer partner
SIRT1: Silent mating type information regulation 2 homolog 1
SRC: Protooncogene tyrosine-protein kinase
SREBP: Sterol regulatory element-binding protein
STING: An ER adaptor, the stimulator of interferon genes
Stat3: Signal transducer and activator of transcription 3
TGF*α*: Transforming growth factor *α*
TLR4: Toll-like receptor 4
TRB3: A mammalian homolog of* Drosophila* tribbles functions as a negative modulator of protein kinase B (PKB)
TRAF2: TNF receptor-associated factor 2
TUDCA: Tauroursodeoxycholate
UPR: The unfolded protein response
VEGF: Vascular endothelial growth factor
XBP1: X-box binding protein 1.

## References

[1] Walter P, Ron D (2011). The unfolded protein response: from stress pathway to homeostatic regulation. *Science*.

[2] Cao SS, Kaufman RJ (2013). Targeting endoplasmic reticulum stress in metabolic disease. *Expert Opinion on Therapeutic Targets*.

[3] Fu S, Watkins SM, Hotamisligil GS (2012). The role of endoplasmic reticulum in hepatic lipid homeostasis and stress signaling. *Cell Metabolism*.

[4] Henkel A, Green RM (2013). The unfolded protein response in Fatty liver disease. *Seminars in Liver Disease*.

[5] Wolff S, Weissman JS, Dillin A (2014). Differential scales of protein quality control. *Cell*.

[6] Ozcan L, Tabas I (2012). Role of endoplasmic reticulum stress in metabolic disease and other disorders. *Annual Review of Medicine*.

[7] Kitamura M (2013). The unfolded protein response triggered by environmental factors. *Seminars in Immunopathology*.

[8] Ji C (2008). Dissection of endoplasmic reticulum stress signaling in alcoholic and non-alcoholic liver injury. *Journal of Gastroenterology and Hepatology*.

[9] Ji C (2012). Mechanisms of alcohol-induced endoplasmic reticulum stress and organ injuries. *Biochemistry Research International*.

[10] Kaphalia L, Boroumand N, Hyunsu J, Kaphalia BS, Calhoun WJ (2014). Ethanol metabolism, oxidative stress, and endoplasmic reticulum stress responses in the lungs of hepatic alcohol dehydrogenase deficient deer mice after chronic ethanol feeding. *Toxicology and Applied Pharmacology*.

[11] Ji C, Kaplowitz N (2003). Betaine decreases hyperhomocysteinemia, endoplasmic reticulum stress, and liver injury in alcohol-fed mice. *Gastroenterology*.

[12] Ji C, Chan C, Kaplowitz N (2006). Predominant role of sterol response element binding proteins (SREBP) lipogenic pathways in hepatic steatosis in the murine intragastric ethanol feeding model. *Journal of Hepatology*.

[13] Ji C, Mehrian-Shai R, Chan C, Hsu Y-H, Kaplowitz N (2005). Role of CHOP in hepatic apoptosis in the murine model of intragastric ethanol feeding. *Alcoholism: Clinical and Experimental Research*.

[14] Ji C, Shinohara M, Kuhlenkamp J, Chan C, Kaplowitz N (2007). Mechanisms of protection by the betaine-homocysteine methyltransferase/ betaine system in HepG2 cells and primary mouse hepatocytes. *Hepatology*.

[15] Ji C, Shinohara M, Vance D (2008). Effect of transgenic extrahepatic expression of betaine-homocysteine methyltransferase on alcohol or homocysteine-induced fatty liver. *Alcoholism: Clinical and Experimental Research*.

[16] Xu J, Lai KKY, Verlinsky A (2011). Synergistic steatohepatitis by moderate obesity and alcohol in mice despite increased adiponectin and p-AMPK. *Journal of Hepatology*.

[17] Shinohara M, Ji C, Kaplowitz N (2010). Differences in betaine-homocysteine methyltransferase expression, endoplasmic reticulum stress response, and liver injury between alcohol-fed mice and rats. *Hepatology*.

[18] Tsuchiya M, Ji C, Kosyk O (2012). Interstrain differences in liver injury and one-carbon metabolism in alcohol-fed mice. *Hepatology*.

[19] Ronis MJ, Korourian S, Blackburn ML, Badeaux J, Badger TM (2010). The role of ethanol metabolism in development of alcoholic steatohepatitis in the rat. *Alcohol*.

[20] Esfandiari F, Medici V, Wong DH (2010). Epigenetic regulation of hepatic endoplasmic reticulum stress pathways in the ethanol-fed cystathionine beta synthase-deficient mouse. *Hepatology*.

[21] Esfandiari F, Villanueva JA, Wong DH, French SW, Halsted CH (2005). Chronic ethanol feeding and folate deficiency activate hepatic endoplasmic reticulum stress pathway in micropigs. *American Journal of Physiology—Gastrointestinal and Liver Physiology*.

[22] Ji C, Kaplowitz N, Lau MY, Kao E, Petrovic LM, Lee AS (2011). Liver-specific loss of glucose-regulated protein 78 perturbs the unfolded protein response and exacerbates a spectrum of liver diseases in mice. *Hepatology*.

[23] Fernandez A, Matias N, Fucho R (2013). ASMase is required for chronic alcohol induced hepaticendoplasmic reticulum stress and mitochondrial cholesterol loading. *Journal of Hepatology*.

[24] Tan TC, Crawford DH, Jaskowski LA (2013). Excess iron modulates endoplasmic reticulum stress-associated pathways in a mouse model of alcohol and high-fat diet-induced liver injury. *Laboratory Investigation*.

[25] Tan TC, Crawford DH, Jaskowski LA (2013). A corn oil-based diet protects against combined ethanol and iron-induced liver injury in a mouse model of hemochromatosis. *Alcoholism: Clinical and Experimental Research*.

[26] Galligan JJ, Smathers RL, Fritz KS, Epperson LE, Hunter LE, Petersen DR (2012). Protein carbonylation in a murine model for early alcoholic liver disease. *Chemical Research in Toxicology*.

[27] Galligan JJ, Smathers RL, Shearn CT (2012). Oxidative stress and the ER stress response in a murine model for early-stage alcoholic liver disease. *Journal of Toxicology*.

[28] Nishitani Y, Matsumoto H (2006). Ethanol rapidly causes activation of JNK associated with ER stress under inhibition of ADH. *FEBS Letters*.

[29] Kao E, Shinohara M, Feng M, Lau MY, Ji C (2012). Human immunodeficiency virus protease inhibitors modulate Ca(2+) homeostasis and potentiate alcoholic stress and injury in mice and primary mouse and human hepatocytes. *Hepatology*.

[30] Tazi KA, Bièche I, Paradis V (2007). In vivo altered unfolded protein response and apoptosis in livers from lipopolysaccharide-challenged cirrhotic rats. *Journal of Hepatology*.

[31] Petrasek J, Iracheta-Vellve A, Csak T (2013). STING-IRF3 pathway links endoplasmic reticulum stress with hepatocyte apoptosis in early alcoholic liver disease. *Proceedings of the National Academy of Sciences of the United States of America*.

[32] Passeri MJ, Cinaroglu A, Gao C, Sadler KC (2009). Hepatic steatosis in response to acute alcohol exposure in zebrafish requires sterol regulatory element binding protein activation. *Hepatology*.

[33] Thakur PC, Stuckenholz C, Rivera MR (2011). Lack of de novo phosphatidylinositol synthesis leads to endoplasmic reticulum stress and hepatic steatosis in cdipt-deficient zebrafish. *Hepatology*.

[34] Tsedensodnom O, Vacaru AM, Howarth DL, Yin C, Sadler KC (2013). Ethanol metabolism and oxidative stress are required for unfolded protein response activation and steatosis in zebrafish with alcoholic liver disease. *Disease Models & Mechanisms*.

[35] Ient B, Edwards R, Mould R, Hannah M, Holden-Dye L, O'Connor V (2012). HSP-4 endoplasmic reticulum (ER) stress pathway is not activated in a C. elegans model of ethanol intoxication and withdrawal. *Invertebrate Neuroscience*.

[36] Boukli NM, Saiyed ZM, Ricaurte M (2010). Implications of ER stress, the unfolded protein response, and pro- and anti-apoptotic protein fingerprints in human monocyte-derived dendritic cells treated with alcohol. *Alcoholism: Clinical and Experimental Research*.

[37] Magne L, Blanc E, Legrand B (2011). ATF4 and the integrated stress response are induced by ethanol and cytochrome P450 2E1 in human hepatocytes. *Journal of Hepatology*.

[38] Howarth DL, Vacaru AM, Tsedensodnom O (2012). Alcohol disrupts endoplasmic reticulum function and protein secretion in hepatocytes. *Alcoholism: Clinical and Experimental Research*.

[39] Longato L, Ripp K, Setshedi M (2012). Insulin resistance, ceramide accumulation, and endoplasmic reticulum stress in human chronic alcohol-related liver disease. *Oxidative Medicine and Cellular Longevity*.

[40] Ramirez T, Longato L, Dostalek M, Tong M, Wands JR, de la Monte SM (2013). Insulin resistance, ceramide accumulation and endoplasmic reticulum stress in experimental chronic alcohol-induced steatohepatitis. *Alcohol and Alcoholism*.

[41] Ramirez T, Tong M, Chen WC, Nguyen QG, Wands JR, de la Monte SM (2013). Chronic alcohol-induced hepatic insulin resistance and ER stress ameliorated by PPAR-*δ*, agonist treatment. *Journal of Gastroenterology and Hepatology*.

[42] Tong M, Longato L, Ramirez T, Zabala V, Wands JR, de la Monte SM (2014). Therapeutic reversal of chronic alcohol-related steatohepatitis with the ceramide inhibitor myriocin. *International Journal of Experimental Pathology*.

[43] Cinti DL, Grundin R, Orrenius S (1973). The effect of ethanol on drug oxidations in vitro and the significance of ethanol-cytochrome P-450 interaction. *Biochemical Journal*.

[44] Lieber CS (1987). Microsomal ethanol-oxidizing system. *Enzyme*.

[45] Lieber CS (2004). The discovery of the microsomal ethanol oxidizing system and its physiologic and pathologic role. *Drug Metabolism Reviews*.

[46] Lieber CS (2005). Pathogenesis and treatment of alcoholic liver disease: progress over the last 50 years. *Roczniki Akademii Medycznej w Białymstoku*.

[47] Barak AJ, Beckenhauer HC, Tuma DJ, Badakhsh S (1987). Effects of prolonged ethanol feeding on methionine metabolism in rat liver. *Biochemistry and Cell Biology*.

[48] Blasco C, Caballería J, Deulofeu R (2005). Prevalence and mechanisms of hyperhomocysteinemia in chronic alcoholics. *Alcoholism: Clinical and Experimental Research*.

[49] Hultberg B, Berglund M, Andersson A, Frank A (1993). Elevated plasma homocysteine in alcoholics. *Alcoholism: Clinical and Experimental Research*.

[50] Lutz UC (2008). Alterations in homocysteine metabolism among alcohol dependent patients—clinical, pathobiochemical and genetic aspects. *Current Drug Abuse Reviews*.

[51] Bleich S, Lenz B, Ziegenbein M (2006). Epigenetic DNA hypermethylation of the HERP gene promoter induces down-regulation of its mRNA expression in patients with alcohol dependence. *Alcoholism: Clinical and Experimental Research*.

[52] Win S, Than TA, Fernandez-Checa JC, Kaplowitz N (2014). JNK interaction with Sab mediates ER stress induced inhibition of mitochondrial respiration and cell death. *Cell Death & Disease*.

[53] Han D, Ybanez MD, Johnson HS (2012). Dynamic adaptation of liver mitochondria to chronic alcohol feeding in mice: biogenesis, remodeling, and functional alterations. *The Journal of Biological Chemistry*.

[54] Inokuchi S, Tsukamoto H, Park E, Liu Z-X, Brenner DA, Seki E (2011). Toll-like receptor 4 mediates alcohol-induced steatohepatitis through bone marrow-derived and endogenous liver cells in mice. *Alcoholism: Clinical and Experimental Research*.

[55] Vandewynckel YP, Laukens D, Geerts A (2013). The paradox of the unfolded protein response in cancer. *Anticancer Research*.

[56] Wang WA, Groenendyk J, Michalak M (2014). Endoplasmic reticulum stress associated responses in cancer. *Biochimica et Biophysica Acta*.

[57] Brown JM, Giaccia AJ (1998). The unique physiology of solid tumors: opportunities (and problems) for cancer therapy. *Cancer Research*.

[58] Giaccia AJ, Brown JM, Wouters B, Denko N, Koumenis C (1998). Cancer therapy and tumor physiology. *Science*.

[59] Lee AS (2014). Glucose-regulated proteins in cancer: molecular mechanisms and therapeutic potential. *Nature Reviews Cancer*.

[60] Weston RT, Puthalakath H (2010). Endoplasmic reticulum stress and BCL-2 family members. *Advances in Experimental Medicine and Biology*.

[61] Wang C, Jiang K, Gao D (2013). Clusterin protects hepatocellular carcinoma cells from endoplasmic reticulum stress induced apoptosis through GRP78. *PLoS One*.

[62] Martinon F (2012). Targeting endoplasmic reticulum signaling pathways in cancer. *Acta Oncologica*.

[63] Koumenis C (2006). ER stress, hypoxia tolerance and tumor progression. *Current Molecular Medicine*.

[64] Fels DR, Koumenis C (2006). The PERK/eIF2alpha/ATF4 module of the UPR in hypoxia resistance and tumor growth. *Cancer Biology & Therapy*.

[65] Blais JD, Addison CL, Edge R (2006). Perk-dependent translational regulation promotes tumor cell adaptation and angiogenesis in response to hypoxic stress. *Molecular and Cellular Biology*.

[66] Rzymski T, Milani M, Pike L (2010). Regulation of autophagy by ATF4 in response to severe hypoxia. *Oncogene*.

[67] Cojocari D, Vellanki RN, Sit B, Uehling D, Koritzinsky M, Wouters BG (2013). New small molecule inhibitors of UPR activation demonstrate that PERK, but not IRE1*α*, signaling is essential for promoting adaptation and survival to hypoxia. *Radiotherapy & Oncology*.

[68] Schewe DM, Aguirre-Ghiso JA (2008). ATF6*α*-Rheb-mTOR signaling promotes survival of dormant tumor cells in vivo. *Proceedings of the National Academy of Sciences of the United States of America*.

[69] Hetz C, Bernasconi P, Fisher J (2006). Proapoptotic BAX and BAK modulate the unfolded protein response by a direct interaction with IRE1*α*. *Science*.

[70] Rodriguez DA, Zamorano S, Lisbona F (2012). BH3-only proteins are part of a regulatory network that control the sustained signalling of the unfolded protein response sensor IRE1*α*. *EMBO Journal*.

[71] Vilner BJ, De Costa BR, Bowen WD (1995). Cytotoxic effects of sigma ligands: sigma receptor-mediated alterations in cellular morphology and viability. *Journal of Neuroscience*.

[72] Hayashi T, Su T-P (2007). Sigma-1 Receptor Chaperones at the ER- Mitochondrion Interface Regulate Ca2+ Signaling and Cell Survival. *Cell*.

[73] Grewal P, Viswanathen VA (2012). Liver cancer and alcohol. *Clinical Liver Disease*.

[74] Warren KR, Murray MM (2013). Alcoholic liver disease and pancreatitis: global health problems being addressed by the US National Institute on Alcohol Abuse and Alcoholism. *Journal of Gastroenterology and Hepatology*.

[75] Friedmann PD (2013). Alcohol use in adults. *The New England Journal of Medicine*.

[76] Gunzerath L, Hewitt BG, Li T-K, Warren KR (2011). Alcohol research: past, present, and future. *Annals of the New York Academy of Sciences*.

[77] Zhu X, Zhang J, Fan W (2013). The rs391957 variant cis-regulating oncogene GRP78 expression contributes to the risk of hepatocellular carcinoma. *Carcinogenesis*.

[78] Winder T, Bohanes P, Zhang W (2011). Grp78 promoter polymorphism rs391957 as potential predictor for clinical outcome in gastric and colorectal cancer patients. *Annals of Oncology*.

[79] Zhu X, Chen L, Fan W (2011). An intronic variant in the GRP78, a stress-associated gene, improves prediction for liver cirrhosis in persistent HBV carriers. *PLoS ONE*.

[80] Zhu X, Chen M-S, Tian L-W (2009). Single nucleotide polymorphism of rs430397 in the fifth intron of GRP78 gene and clinical relevance of primary hepatocellular carcinoma in Han Chinese: risk and prognosis. *International Journal of Cancer*.

[81] Liu S, Li K, Li T (2013). Association between promoter polymorphisms of the GRP78 gene and risk of type 2 diabetes in a Chinese han population. *DNA and Cell Biology*.

[82] Merrick DT (2012). GRP78, intronic polymorphisms, and pharmacogenomics in non-small cell lung cancer. *Chest*.

[83] Lau MY, Han H, Hu J, Ji C (2013). Association of cyclin D and estrogen receptor *α*36 with hepatocellular adenomas of female mice under chronic endoplasmic reticulum stress. *Journal of Gastroenterology and Hepatology*.

[84] Han H, Hu J, Lau MY, Feng M, Petrovic LM, Ji C (2013). Altered methylation and expression of ER-associated degradation factors in long-term alcohol and constitutive ER stress-induced murine hepatic tumors. *Frontiers in Genetics*.

[85] Giannelli G, Napoli N, Antonaci S (2007). Tyrosine kinase inhibitors: a potential approach to the treatment of hepatocellular carcinoma. *Current Pharmaceutical Design*.

[86] Muntané J, De la Rosa AJ, Docobo F, García-Carbonero R, Padillo FJ (2013). Targeting tyrosine kinase receptors in hepatocellular carcinoma. *Current Cancer Drug Targets*.

[87] Bioulac-Sage P, Taouji S, Possenti L, Balabaud C (2012). Hepatocellular adenoma subtypes: the impact of overweight and obesity. *Liver International*.

[88] Nault J-C, Zucman-Rossi J (2011). Genetics of hepatobiliary carcinogenesis. *Seminars in Liver Disease*.

[89] Katabathina VS, Menias CO, Shanbhogue AKP, Jagirdar J, Paspulati RM, Prasad SR (2011). Genetics and imaging of hepatocellular adenomas: 2011 update. *Radiographics*.

[90] Lin H, Van Den Esschert J, Liu C, Van Gulik TM (2011). Systematic review of hepatocellular adenoma in China and other regions. *Journal of Gastroenterology and Hepatology*.

[91] Edmondson HA, Henderson B, Benton B (1976). Liver cell adenomas associated with use of oral contraceptives. *The New England Journal of Medicine*.

[92] Bioulac-Sage P, Cubel G, Balabaud C, Zucman-Rossi J (2011). Revisiting the pathology of resected benign hepatocellular nodules using new immunohistochemical markers. *Seminars in Liver Disease*.

[93] Guichard C, Amaddeo G, Imbeaud S (2012). Integrated analysis of somatic mutations and focal copy-number changes identifies key genes and pathways in hepatocellular carcinoma. *Nature Genetics*.

[94] Brewer JW, Hendershot LM, Sherr CJ, Diehl JA (1999). Mammalian unfolded protein response inhibits cyclin D1 translation and cell-cycle progression. *Proceedings of the National Academy of Sciences of the United States of America*.

[95] Brewer JW, Diehl JA (2000). PERK mediates cell-cycle exit during the mammalian unfolded protein response. *Proceedings of the National Academy of Sciences of the United States of America*.

[96] Hamanaka RB, Bennett BS, Cullinan SB, Diehl JA (2005). PERK and GCN2 contribute to eIF2*α* phosphorylation and cell cycle arrest after activation of the unfolded protein response pathway. *Molecular Biology of the Cell*.

[97] Kim JK, Diehl JA (2009). Nuclear cyclin D1: an oncogenic driver in human cancer. *Journal of Cellular Physiology*.

[98] Pestell RG (2013). New roles of cyclin D1. *The American Journal of Pathology*.

[99] Musgrove EA, Caldon CE, Barraclough J, Stone A, Sutherland RL (2011). Cyclin D as a therapeutic target in cancer. *Nature Reviews Cancer*.

[100] Fu M, Wang C, Li Z, Sakamaki T, Pestell RG (2004). Minireview: cyclin D1: normal and abnormal functions. *Endocrinology*.

[101] Oyama T, Kashiwabara K, Yoshimoto K, Arnold A, Koerner F (1998). Frequent overexpression of the cyclin D1 oncogene in invasive lobular carcinoma of the breast. *Cancer Research*.

[102] Shoker BS, Jarvis C, Davies MPA, Iqbal M, Sibson DR, Sloane JP (2001). Immunodetectable cyclin D1 is associated with oestrogen receptor but not Ki67 in normal, cancerous and precancerous breast lesions. *British Journal of Cancer*.

[103] Ledda-Columbano GM, Pibiri M, Concas D, Cossu C, Tripodi M, Columbano A (2002). Loss of cyclin D1 does not inhibit the proliferative response of mouse liver to mitogenic stimuli. *Hepatology*.

[104] Lu JW, Lin YM, Chang JG (2013). Clinical implications of deregulated CDK4 and Cyclin D1 expression in patients with human hepatocellular carcinoma. *Medical Oncology*.

[105] Suh JH, Shenvi SV, Dixon BM (2004). Decline in transcriptional activity of Nrf2 causes age-related loss of glutathione synthesis, which is reversible with lipoic acid. *Proceedings of the National Academy of Sciences of the United States of America*.

[106] Zhang Y, Wang L (2011). Nuclear receptor small heterodimer partner in apoptosis signaling and liver cancer. *Cancers*.

[107] Zhang Y, Hagedorn CH, Wang L (2011). Role of nuclear receptor SHP in metabolism and cancer. *Biochimica et Biophysica Acta*.

[108] Zhang W, Hietakangas V, Wee S, Lim SC, Gunaratne J, Cohen SM (2013). ER stress potentiates insulin resistance through PERK-mediated FOXO phosphorylation. *Genes & Development*.

[109] Czaja MJ, Ding WX, Donohue TM (2013). Functions of autophagy in normal and diseased liver. *Autophagy*.

[110] Tikhanovich I, Kuravi S, Campbell RV (2014). Regulation of FOXO3 by phosphorylation and methylation in hepatitis C virus infection and alcohol exposure. *Hepatology*.

[111] Kopycinska J, Kempińska-Podhorodecka A, Haas T (2013). Activation of FoxO3a/Bim axis in patients with Primary Biliary Cirrhosis. *Liver International*.

[112] Wu T, Zhao F, Gao B (2014). Hrd1 suppresses Nrf2-mediated cellular protection during liver cirrhosis. *Genes & Development*.

[113] Yan Z, Tan W, Dan Y (2012). Estrogen receptor alpha gene polymorphisms and risk of HBV-related acute liver failure in the Chinese population. *BMC Medical Genetics*.

[114] Miceli V, Cocciadiferro L, Fregapane M (2011). Expression of wild-type and variant estrogen receptor alpha in liver carcinogenesis and tumor progression. *OMICS A Journal of Integrative Biology*.

[115] Quaynor SD, Stradtman Jr EW, Kim HG (2013). Delayed puberty and estrogen resistance in a woman with estrogen receptor *α* variant. *The New England Journal of Medicine*.

[116] Clegg DJ, Palmer BF (2013). Effects of an estrogen receptor *α* variant. *The New England Journal of Medicine*.

[117] Moeini A, Cornellà H, Villanueva A (2012). Emerging signaling pathways in hepatocellular carcinoma. *Liver Cancer*.

[118] Vranic S, Gatalica Z, Deng H (2011). ER-*α*36, a novel isoform of ER-*α*66, is commonly over-expressed in apocrine and adenoid cystic carcinomas of the breast. *Journal of Clinical Pathology*.

[119] Chaudhri RA, Olivares-Navarrete R, Cuenca N, Hadadi A, Boyan BD, Schwartz Z (2012). Membrane estrogen signaling enhances tumorigenesis and metastatic potential of breast cancer cells via estrogen receptor-*α*36 (ER*α*36). *Journal of Biological Chemistry*.

[120] Wang J, Li J, Fang R, Xie S, Wang L, Xu C (2012). Expression of ER*α*36 in gastric cancer samples and their matched normal tissues. *Oncology Letters*.

[121] Zhang XT, Ding L, Kang LG, Wang ZY (2012). Involvement of ER-*α*36, Src, EGFR and STAT5 in the biphasic estrogen signaling of ER-negative breast cancer cells. *Oncology Reports*.

[122] Chaudhri RA, Olivares-Navarrete R, Cuenca N, Hadadi A, Boyan BD, Schwartz Z (2012). Membrane estrogen signaling enhances tumorigenesis and metastatic potential of breast cancer cells via estrogen receptor-*α*36 (ER*α*36). *Journal of Biological Chemistry*.

[123] Di Maio M, Daniele B, Pignata S (2008). IS human hepatocellular carcinoma a hormone-responsive tumor?. *World Journal of Gastroenterology*.

[124] Center MM, Jemal A (2011). International trends in liver cancer incidence rates. *Cancer Epidemiology Biomarkers and Prevention*.

[125] Venook AP, Papandreou C, Furuse J, de Guevara LL (2010). The incidence and epidemiology of hepatocellular carcinoma: a global and regional perspective. *The Oncologist*.

